# Cancer and Associated Therapies Impact the Skeletal Muscle Proteome

**DOI:** 10.3389/fphys.2022.879263

**Published:** 2022-05-27

**Authors:** Dillon E. L., Wright T. J., Filley A. R., Pulliam A. B., Randolph K. M., Danesi C. P., Gilkison C. R., Wiktorowicz J. E., Soman K. V., Urban R. J., Sheffield-Moore M

**Affiliations:** ^1^ Department of Internal Medicine, University of Texas Medical Branch, Galveston, TX, United States; ^2^ Department of Health and Kinesiology, Texas A&M University, College Station, TX, United States; ^3^ Department of Biomedical Sciences, Texas A&M University, College Station, TX, United States

**Keywords:** cachexia, atrophy, post-translational modifications, phosphorylation, nitrosylation, proteome, testosterone

## Abstract

**Introduction:** Both cancer and cancer associated therapies (CAT; including chemotherapy or concurrent chemoradiation) disrupt cellular metabolism throughout the body, including the regulation of skeletal muscle mass and function. Adjunct testosterone therapy during standard of care chemotherapy and chemoradiation modulates CAT-induced dysregulation of skeletal muscle metabolism and protects lean body mass during CAT. However, the extent to which the skeletal muscle proteome is altered under these therapeutic conditions is unknown.

**Objective:** We probed the skeletal muscle proteome of cancer patients as an ancillary analysis following a randomized, double-blind, placebo-controlled phase II trial investigating the effect of adjunct testosterone on body composition in men and women with advanced cancers undergoing CAT.

**Methods:** Men and women diagnosed with late stage (≥IIB) or recurrent head and neck or cervical cancer who were scheduled to receive standard of care CAT were administered an adjunct 7 weeks treatment of weekly intramuscular injections of either 100 mg testosterone (CAT+T, *n* = 7; 2M/5F) or placebo/saline (CAT+P, *n* = 6; 4M/2F). Biopsies were performed on the vastus lateralis before (PRE) and after (POST) the 7 weeks treatment. Extracted proteins were separated with 2-dimensional gel electrophoresis (2DE), and subjected to analyses of total protein abundance, phosphorylation and S-nitrosylation. Proteoforms showing significant 1.5 fold differences (*t*-test *p* ≤ 0.05) between PRE and POST timepoints were identified by mass spectroscopy (MS), and lists of altered proteins were subjected to Gene Set Enrichment Analysis (GSEA) to identify affected pathways.

**Results:** A total of 756 distinct protein spots were identified. Of those spots, 102 were found to be altered in terms of abundance, phosphorylation, or S-nitrosylation, and identified by mass spectroscopy analysis to represent 58 unique proteins. Among the biological processes and pathways identified, CAT+P predominantly impacted metabolic processes, cell assembly, oxygen transport, and apoptotic signaling, while CAT+T impacted transcription regulation, muscle differentiation, muscle development, and contraction.

**Conclusion:** Cancer and CAT significantly altered the skeletal muscle proteome in a manner suggestive of loss of structural integrity, reduced contractile function, and disrupted metabolism. Proteomic analysis suggests that the addition of adjunct testosterone minimized the structural and contractile influence of cancer and its associated therapies.

## Introduction

Cancer progression is often associated with cachexia, a debilitating, and phenotypically recognizable loss in body mass and physical function. In comparison to several other common catabolic conditions including prolonged physical inactivity and sarcopenia of aging, cancer cachexia is a mechanically distinct multisystem wasting condition that extends beyond the typical losses in muscle mass and strength. Cancer cachexia is characterized by wasting of organs and tissues that includes atrophy of skeletal muscle mass as well as adipose tissue, whereas conditions such as sarcopenia of aging or sedentary lifestyles are typically accompanied by increased adiposity along with more gradual declines in skeletal mass (Ali and Garcia, 2014).

Distinctions between catabolic conditions have been observed at the molecular level, indicating that while various conditions can disrupt skeletal muscle proteostasis and trigger skeletal muscle atrophy, the catabolic triggers have distinct proteomic patterns (Hunt et al., 2021). Genomic and proteomic analyses of mouse muscle by Hunt et al. support the current understanding that more acute drivers of atrophy such as corticosteroids and cachexia are characterized by decreased protein synthesis and increased degradation while age related muscle loss involves reduced maintenance of protein quality but not necessarily increased protein degradation (Hunt et al., 2021). Characterizing and comparing the differences and similarities in the skeletal muscle proteome between distinct wasting conditions will help provide insight into the mechanisms that drive muscle atrophy and inform development of targeted interventions against cachexia (Peixoto da Silva et al., 2020). Given the frequency of cachexia in people with cancer it is important to understand the proteomic profile of skeletal muscle in this population, including how interventions affect the abundance and posttranslational modification of specific proteins as well as the pathways associated with these changes. Confounding factors (including but not limited to the type of cancer, recurrence or progression of the disease, type or length of standard of care cancer treatment, patient age, and sex of the patient) which affect the progression of cancer cachexia, are not always well-controlled and can vary immensely.

Key intervention strategies against losses of muscle mass and strength encountered as a consequence of sedentary lifestyles, and even sarcopenia, include modulation of diet and physical activity. While immensely important as part of the therapeutic approach during cancer treatment, these interventions alone have not been proven to be adequate in countering the progression of cachexia (Schütte et al., 2020; Kasvis and Kilgour, 2021; Kim et al., 2021; van der Meij et al., 2021). In addition, cancer associated therapies (CAT) including chemo and radiation, inevitably add to the complexity when exploring the mechanisms that drive cancer cachexia as these treatments are themselves physiologically disruptive to cellular processes (Damrauer et al., 2018; Pin et al., 2019). Thus, while these treatments are clinically beneficial for targeting cancerous cells, improving patient quality of life, and aiding survival, the treatment administered to the patient becomes part of the cellular milieu and may affect key biological processes.

Hormones, including androgens such as testosterone which are widely known for the anabolic effect on skeletal muscle, have proven clinical efficacy with treatment approaches demonstrated in male as well as female populations (Sheffield-Moore et al., 2006; Horstman et al., 2012; Dos Santos and Storer, 2022). However, testosterone treatment as an intervention against muscle wasting in cancer patients is novel. We recently investigated the effect of adjunct testosterone on body composition and quality of life in men and women undergoing CAT for late-stage squamous cell carcinoma of the cervix or head and neck (Wright et al., 2018). This National Cancer Institute (NCI) funded, randomized, double-blind, placebo-controlled phase II trial demonstrated that 7 weeks of testosterone supplementation ameliorated weight loss in late stage cancer patients by preferentially increasing lean body mass. The improvements in lean body mass were associated with clinically meaningful improvements in patient quality of life and physical performance, although adjuvant testosterone therapy did not increase overall patient survival. To better understand how the course of standard of care CAT with or without adjuvant testosterone treatment differentially alters the skeletal muscle proteome, we compared skeletal muscle biopsies from the patients before and after respective treatment.

## Methods

### Patients/Subjects

The study complied with the Declaration of Helsinki and was approved by The University of Texas Medical Branch (UTMB) Institutional Review Board (IRB). Written informed consent was obtained from all subjects prior to being studied at the ITS-CRC (Institute for Translational Sciences - Clinical Research Center) at UTMB. Details of the study design and primary outcomes from this intent-to-treat investigation have been published previously (Wright et al., 2018) and the data are made available at https://doi.org/10.18738/T8/RFRQE2. Only subjects with both pre and post treatment muscle tissue samples available for proteomics were included in these ancillary analyses. These analyses include data from 13 squamous cell carcinoma patients enrolled in a double-blinded testosterone vs. placebo treatment study funded by the NCI and was designed to not interfere with standard of care treatment. Volunteers included patients with advanced or recurrent squamous cell carcinoma of the cervix (stages IIB, IIIA, and IIIB) between the ages of 18 and 65, and patients with advanced (stage III or IV) or recurrent head and neck squamous cell carcinoma between the ages of 18 and 75 (Table 1). The CAT+T group includes three cervical cancer patients and four head and neck cancer patients. One cervical cancer patient also followed an amino acid supplementation regimen in addition to receiving CAT+T treatment and POST study data collection occurred 3 months after PRE study data collection due to interruptions and postponements in her treatment schedule. Because of the intent-to-treat nature of the investigation, this patient was not excluded from participation or analysis. Pre-post analyses for CAT+T include five female and two male patients (age 53.6 ± 5.1 years, BMI 21.7 ± 8.0 kg/m^2^). Tissues from four CAT+T subjects were available for S-nitrosylation analyses. The CAT+P group includes two cervical cancer patients and four head and neck cancer patients. Tissues from four CAT+P subjects were included in S-nitrosylation analyses. Pre-post analyses for CAT+P group include two female and four male patients (age 49.7 ± 10.3 years, BMI 25.9 ± 8.0 kg/m^2^).

**TABLE 1:** Patient population. Baseline demographic information for cancer patients receiving either cancer associated therapy with adjunct testosterone (CAT+T) or placebo (CAT+P). Based on muscle biopsy tissue availability, samples were analyzed for protein abundance, phosphorylation, and S-nitrosylation. H&N, Head and Neck.

								Analyses
#	Cancer type	CAT+T/CAT+P	Female/Male	Age (years)	Height (cm)	Weight (kg)	Baseline testosterone (ng/dl)	Abundance phosphorylation S-Nitrosylation
1	Cervical	CAT+T^a^	F	49	154.9	42.6	15.6	A,P
2	Cervical	CAT+T	F	48	164.2	51.3	14	A,P
3	Cervical	CAT+T	F	53	164.0	99.1	13	A,P
4	H&N	CAT+T	F	51	162.0	40.8	5	A,P,N
5	H&N	CAT+T	F	53	164.0	40.5	47	A,P,N
6	H&N	CAT+T	M	60	177.0	78.2	252	A,P,N
7	H&N	CAT+T	M	61	170.5	65.0	450	A,P,N
8	Cervical	CAT+P	F	49	163.0	52.0	11	A,P
9	Cervical	CAT+P	F	47	164.0	50.9	8	A,P,N
10	H&N	CAT+P	M	48	180.1	130.6	100	A,P
11	H&N	CAT+P	M	35	171.0	85.6	449	A,P,N
12	H&N	CAT+P	M	67	184.5	72.8	110	A,P,N
13	H&N	CAT+P	M	52	160.2	62.0	623	A,P,N

a Subject also received amino acid supplementation.

### Testosterone Treatment

The testosterone treatment followed a common replacement schedule used to treat hypogonadal men. Patients received weekly intramuscular injections of either 100 mg testosterone enanthate or sterile saline (placebo) over a period of 7 weeks. Testosterone and placebo injections were given by a nurse using an opaque syringe to obscure visual differences between testosterone and placebo. Because of the late stages of cancer examined in this study, both men and women were treated with the same dose of testosterone as this dose and duration would not be expected to affect female secondary sexual characteristics.

### Muscle Biopsy Procedure

Muscle tissue was collected before (PRE) and after (POST) completion of standard of care chemo/radiation therapy during which time the patients were randomized to also receive either placebo (CAT+P) or testosterone (CAT+T) treatment. Muscle biopsy procedures were performed as described elsewhere (5, 6). Briefly, a site was marked over the vastus lateralis and cleaned with Betadine. Lidocaine (1%) was administered to the skin and muscle. An approximately 5 mm incision was made through the skin and fascia and a 5 mm Bergström needle was advanced into the muscle. While suction was applied, 100–200 mg of skeletal muscle tissue was collected by opening and closing the cutting window of the biopsy needle. The incision was sutured and covered with Bacitracin and steri-strips. Ice was applied to the site and ibuprofen was provided to the subject to alleviate soreness.

### Skeletal Muscle Proteomics

Abundance and Phosphorylation - Proteomic analyses were performed by the UTMB Biomolecular Resource Facility (BRF) in fractionated muscle extracts using a Biofluids Analytical Platform (BAP) as previously described (Sheffield-Moore et al., 2013; Dillon et al., 2019). These analyses were completed in one continuous effort once all the muscle samples had been collected. The BAP fractionation component combines Superdex S-75 size-exclusion chromatography (SEC) of biofluids with electronically triggered fraction collection to create protein and peptide pools for subsequent separation and analysis. Fractionated samples were subjected to 2D gel electrophoresis (2DE) and stained for phosphoproteins (ProQ Diamond dye) or total proteins (Sypro Ruby dye). The gels were imaged, and analyzed using SameSpots 2D Gel Image Analysis Software for Differential Expression (TotalLab, Newcastle upon Tyne, United Kingdom). Images were aligned using select reference spots and log-transformed spot intensities were quantitatively compared between timepoints within groups (PRE vs. POST). Spots with significant changes (uncorrected *p* ≤ 0.05 and absolute fold differences ≥1.50) between PRE and POST time points identified by SameSpots within either group (CAT+P or CAT+T) were identified by Matrix Assisted Laser Desorption Ionization Time of Flight Mass Spectrometry (MALDI TOF MS), and compared against the Uniprot protein database for identification.

Nitrosylation—Analyses of the S-nitrosoproteome were performed using a fluorescence saturation technique (SNOFlo) separately from the other skeletal muscle proteome analyses (abundances and phosphorylation) as described previously using a ratio of ratios method (Wiktorowicz et al., 2011; Sheffield-Moore et al., 2013; Wiktorowicz et al., 2017). Briefly, after quantification of cysteine content (cysteic acid), denatured sample aliquots were split and either labeled with Bodipy Fl-maleimide (BD) or treated with ascorbate to reverse S-nitrosylation in the samples. Both sets of samples were dialyzed against denaturing buffer followed by BD labeling of the ascorbate treated samples. After two-dimensional gel electrophoresis, spot quantification by fluorescence imaging, and image analysis, protein abundance was measured by calculating the ratio of spot volumes on PRE versus POST gels of the ascorbate-treated samples. The ratios for the samples not treated with ascorbate were calculated similarly. The levels of S-nitrosylation were then expressed as the ratio of the two ratios (ratio of ratios), with the non-ascorbate-treated ratios (representing the sum of S-nitrosylated and abundance differences) normalized against the ascorbate-treated ratios (abundance difference only). A negative ratio of ratios indicates increased cysteinyl S-nitrosylation, and a positive ratio of ratios decreased S-nitrosylation.

### Data and Statistical Analyses

To identify potential biological processes and molecular pathways that were altered within each group, the sets of significantly altered protein IDs from each grouping were subjected to gene set enrichment analysis (GSEA) based on the 2021 Gene Ontology (GO) and Kyoto Encyclopedia of Genes and Genomes (KEGG) databases using the online Enrichr platform (https://amp.pharm.mssm.edu/Enrichr/) (Chen et al., 2013; Kuleshov et al., 2016; Xie et al., 2021). Significantly altered biological pathways were identified by an adjusted *p* < 0.05 using the Fisher exact test, and Benjamini–Hochberg method for correction for multiple hypotheses testing. Data were visualized based on an Enrichr combined score calculated by multiplying the log of the *p*-value from the Fisher exact test by the z-score of the deviation from the expected rank. Separate analyses were performed to identify altered pathways based on significantly altered protein abundance, S-nitrosylation, and phosphorylation. The processes and pathways identified through Enricher were interpreted in context of the phenotypical changes observed in the placebo vs. testosterone treated groups as previously reported (Wright et al., 2018).

## Results

### Patients

Changes in patient body composition and strength were main outcomes of the original study and details have been reported previously (Wright et al., 2018). Briefly, total body weight decreased in the placebo treated group and remained unchanged during testosterone treatment. Lean mass decreased with placebo treatment while this increased with testosterone treatment. Fat mass decreased in both the placebo and testosterone groups. There were no significant changes in muscle strength or physical performance in either group. There were no differences in 1-year survival between the groups.

### Proteomics

**FIGURE 1:**
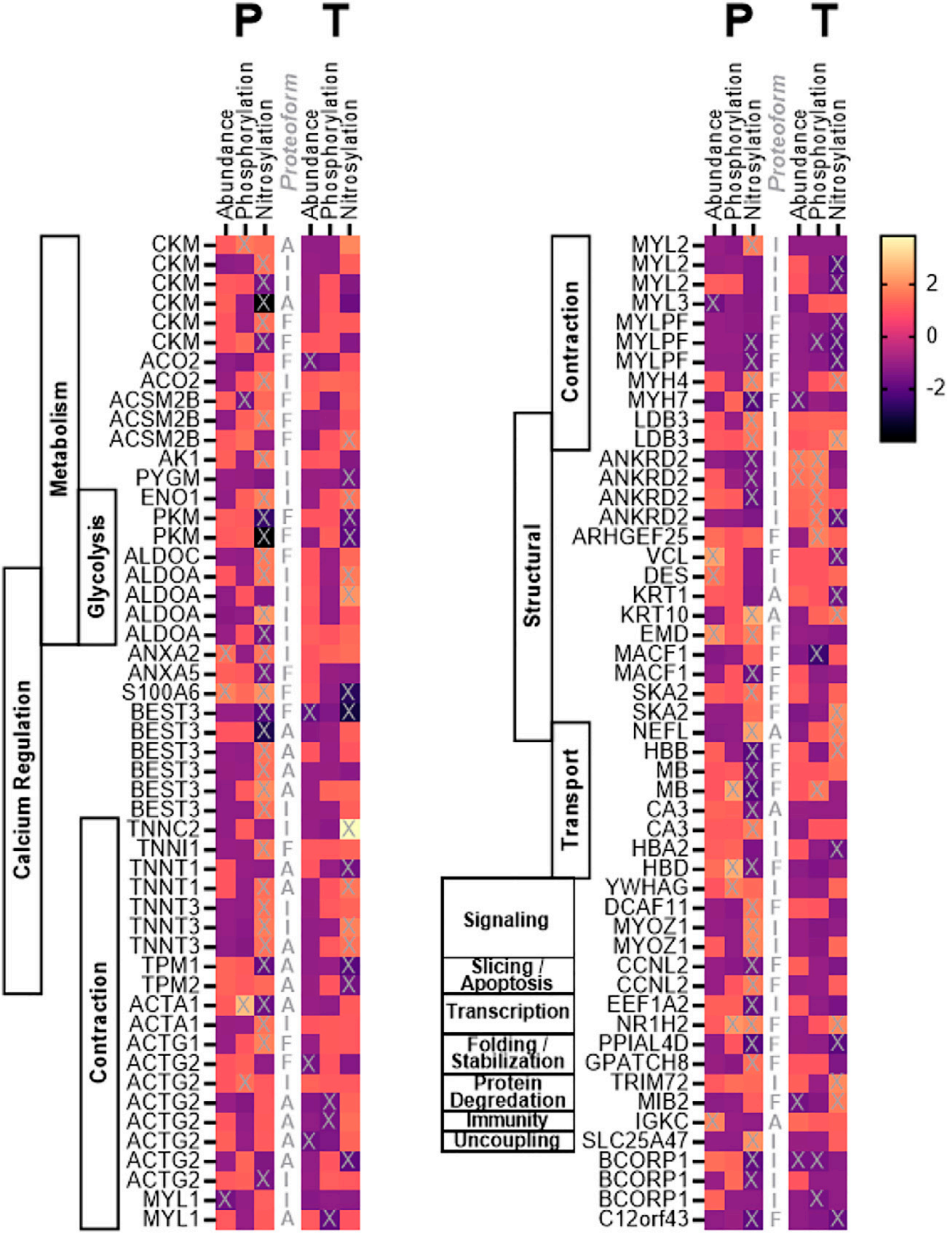
Heat map. Heat map depicting the fold change in abundance, phosphorylation, and S-nitrosylation of skeletal muscle proteins in cancer patients following 7 weeks of treatment with testosterone (T) or placebo (P). Proteoforms marked with “X” changed significantly (*p* < 0.05) from pre to post treatment. Proteoforms are labeled (central column) as either intact (I), fragments (F), or aggregate (A) based on identification and predicted molecular weight as described in the text.

Of 756 distinct spots, 102 were determined to be significantly altered (>1.50 fold change AND *p* < 0.05) from pre to post treatment in abundance (Placebo, *n* = 7 spots; Testosterone, *n* = 8 spots), phosphorylation (Placebo, n = 6 spots; Testosterone, *n* = 7 spots), and/or S-nitrosylation (Placebo, *n* = 59 spots; Testosterone, *n* = 26 spots), and identified using MS. These significantly altered spots were identified by MS, and found to represent 58 unique proteins (Figure 1). To facilitate interpretation, the spots were categorized based on the predicted molecular weights (MW) published in the UNIPROT database (www.uniprot.org). Of the 102 spots, 41 spots reflected proteoforms with MWs that corresponded to full-size proteins (within 15% of predicted UNIPROT MW), 22 spots were assumed to reflect protein dimers and aggregates (exceeded the predicted MW by at least 15%), and 39 spots were assumed to be fragments (more than 15% below predicted MW).

### Protein Abundance Changes

In the CAT+P (placebo) group, cancer and related therapy was associated with significant changes (POST vs. PRE) in the abundance of seven proteoforms including four intact proteins, one aggregate, and two fragments (Figure 1, Supplementary Table S1). Significantly altered intact proteins included those involved with muscle contraction (MYL3, MYL1), cellular structure (DES), and calcium regulation (ANXA2). Of the intact proteoforms that changed, MYL3 and MYL1 decreased in abundance while DES and ANXA2 increased in abundance. Cancer combined with standard of care therapy plus testosterone treatment (CAT+T) was associated with changes in abundance of two intact proteoforms, one aggregate, and five fragments (Figure 1). Altered intact proteins included increased abundance of the structural protein ANKRD2 and decreased abundance of BCORP1.

### Protein Phosphorylation Changes

The course of cancer plus standard of care treatment (CAT+P) led to significant changes in phosphorylation of two intact proteoforms, two fragments, and two aggregates (Figure 1, Supplementary Table S2). Increased phosphorylation occurred in intact proteoforms of the signaling protein YWHAG and the contractile myofilament protein ACTG2. In the CAT+T group, phosphorylation of six intact proteoforms, four fragments, and three aggregates changed significantly during the course of treatment. Altered intact proteoforms included increased phosphorylation of ANKRD2 (4 spots) and decreased phosphorylations in BCORP (2 spots). Phosphorylation changes in these two proteins coincided with similar changes in abundance in these groups.

### Protein S-Nitrosylation Changes

In the CAT+P group, a significant change in S-nitrosylation was seen in 26 intact proteoforms, 11 aggregates, and 22 fragments (Figure 1, Supplementary Table S3). The intact proteins included metabolic proteins ALDOA (3 spots), ENO1, CKM (2 spots), AK1, and ACO2, transport protein CA3, transcriptional protein EEF1A2, structural proteins ANKRD2 (3 spots) and LDB3, contractile proteins TNNT3 (2 spots), MYL2, ACTA1, and ACTG2, signaling protein MYOZ1 (2 spots), calcium regulation proteins ANXA2 and BEST3, uncoupling protein SLC25A47, and protein with uncertain function BCORP1 (2 spots). In the CAT+T group, the proteins that were nitrosylated differently after treatment included 10 intact proteoforms, nine fragments, and seven aggregates. The intact proteins included metabolic proteins ALDOA, PYGM, and ENO1, structural proteins ANKRD2 and LDB3, contractile proteins MYL2 (2 spots) and TNNC2, transport protein HBA2 and degradation protein TRIM72. S-Nitrosylation of ALDOA, ENO1, ANKRD2, LDB3, and MYL2 changed significantly in both CAT+P and CAT+T. Overall, 36 proteoforms were upregulated in CAT+P and 23 proteoforms were downregulated over the course of treatment. In CAT+T, 16 proteoforms were downregulated and 11 were upregulated.

### Gene Set Enrichment Analysis

**FIGURE 2:**
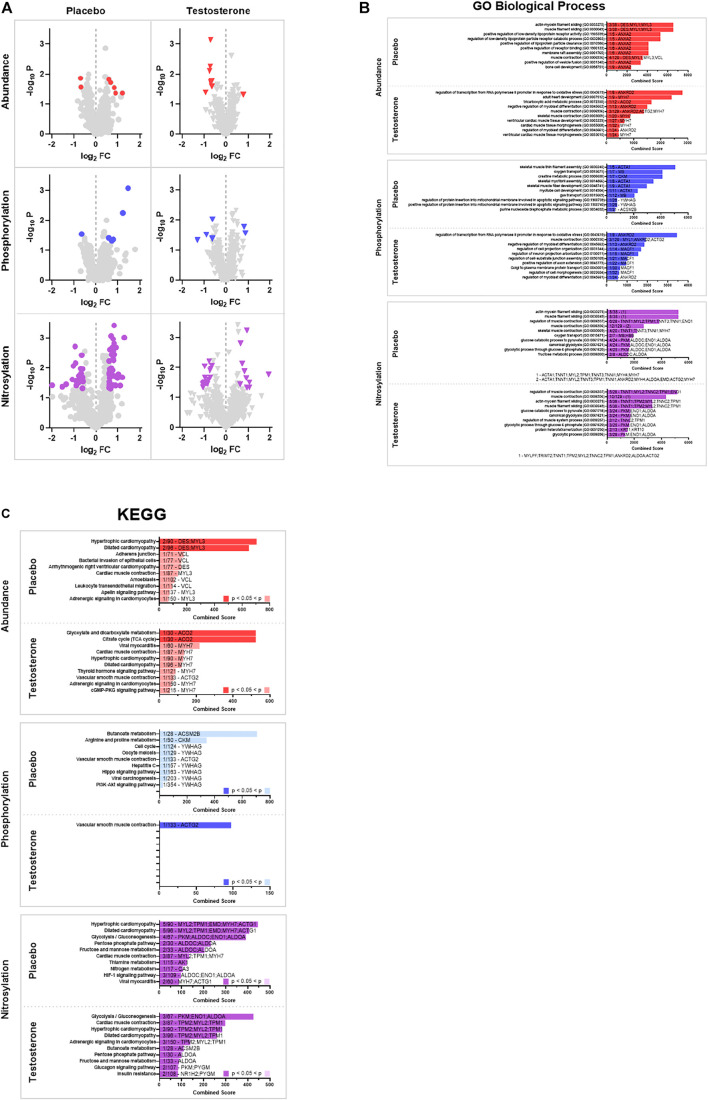
Gene set enrichment analysis (GSEA). **(A)** Volcano Plots. Volcano plots of protein spots identified by 2D electrophoresis represented as -log10 *p*-value vs. log2 of fold change from pre to post. Proteins were deemed significantly altered with a *p* < 0.05 and a log2 fold change >1.5 (increased) or < −1.5 (decreased). Analyses identified significant differences in abundance (red), phosphorylation (blue), and S-nitrosylation (purple) between pre and post treatment biopsies for a subset of proteins (significantly altered proteins are colored). **(B)** Gene Ontology (GO). Significantly altered proteins were used to identify affected gene sets using the 2021 GO Biological Process 2021 database. The top 10 pathways identified by Enrichr combined score (see text for description) are displayed. Pathways that were identified as significantly altered (adjusted *p* < 0.05) are denoted by a dark colored bar for abundance (red), phosphorylation (blue), and S-nitrosylation (purple). The number and identity of significantly altered proteins and total number of proteins in the identified pathway are denoted on each bar. **(C)** Kyoto Encyclopedia of Genes and Genomes (KEGG). Significantly altered proteins were used to identify affected pathways using the 2021 KEGG pathway database. The top 10 pathways identified by Enrichr combined score (see text for description) are displayed. Pathways that were identified as significantly altered (adjusted *p* < 0.05) are denoted by a dark colored bar for abundance (red), phosphorylation (blue), and S-nitrosylation (purple). The number and identity of significantly altered proteins and total number of proteins in the identified pathway are denoted on each bar.

Cancer and CAT with or without testosterone supplementation resulted in significant changes in the abundance and post-translational modifications (phosphorylation and S-nitrosylation) of numerous proteins within the skeletal muscle proteome. Because the net effects of individual protein modifications on either the up or down regulation of entire processes and pathways are complex or unknown, the significantly altered proteins (positive and negative fold changes in intact, fragmented, or aggregate proteoforms) were assessed together to identify affected gene sets (GO) and pathways (KEGG). Analyses of the proteomic responses in vastus lateralis from advanced cancer patients receiving CAT with or without adjuvant testosterone treatment revealed several muscle specific gene sets (GO, Figures 2A,B, Supplementary Figure S1) and pathways (KEGG, Figures 2A,C, Supplementary Figure S2) that were affected as demonstrated by changes in protein abundances, phosphorylation, and S-nitrosylation of key skeletal muscle regulators. Gene set (GO) analyses indicated that the course of CAT+P (control group) had protein abundance changes associated with muscle filament sliding, lipoprotein regulation, and cell membrane processes such as raft assembly and vesicle fusion. Muscle contraction was identified as an altered process regardless of treatment with testosterone or placebo. Additionally, treatment with testosterone (CAT+T) redirected cellular processes towards regulation of proteins involved in cell development, morphogenesis, and cell differentiation. Pathway analyses (KEGG) indicated that CAT+P altered abundances of proteins involved in myopathy related pathways while CAT+T affected cell metabolism pathways. Protein phosphorylation changes during CAT+P were largely directed at metabolic processes, oxygen transport, mitochondria apoptotic signaling, and skeletal muscle development. There were no shared biological processes identified between CAT+P and CAT+T based on phosphorylation changes. The effects of testosterone treatment on protein phosphorylation were in line with the changes in protein abundances. Testosterone treatment appeared to affect regulation of cell differentiation, morphogenesis, and assembly. Pathway analyses (KEGG) indicated that CAT+P marginally affected phosphorylation of proteins involved in butanoate and amino acid metabolism. Conversely, CAT+T modified smooth muscle contraction pathway proteins. The biological processes identified by changes in S-nitrosylation were fairly consistent between CAT+P and CAT+T suggesting that cancer and CAT may have been the predominant regulators of S-nitrosylation. Over the course of CAT, regardless of treatment with testosterone, S-nitrosylation changes appeared largely directed towards processes associated with regulation of muscle contraction and energy metabolism. Pathway analyses (KEGG) indicated that CAT+P affected S-nitrosylation of proteins involving numerous pathways related to myopathies and cell metabolism. CAT+T affected the same pathways identified in CAT+P but also affected proteins with roles in butanoate metabolism, adrenergic signaling, and insulin resistance pathways.

## Discussion

Seven weeks of cancer and standard-of-care cancer treatment was associated with significantly altered skeletal muscle protein abundance, phosphorylation, and S-nitrosylation. Although adjunct testosterone treatment largely preserved skeletal muscle mass in these patients (Wright et al., 2018), proteostasis was disrupted both with and without adjunct testosterone. Supporting these observations, treatment with testosterone likely promoted several biological processes involved in muscle tissue development, including cell differentiation and morphogenesis. The most noticeable differences between placebo and testosterone treated muscle included protein abundance and phosphorylation. None of the same proteoforms changed in abundance when comparing these two groups. Cancer and CAT profoundly affected protein S-nitrosylation regardless of testosterone treatment. The pathways identified as being most altered tended to converge around myopathies, muscle contraction (including smooth and cardiac muscle), and substrate metabolism. The changes in the skeletal muscle proteomes support the phenotypic changes, such as preserved muscle mass in the CAT+T compared to the CAT+P group, that were previously reported in these patients (Wright et al., 2018).

Skeletal muscle atrophy, as seen in cancer cachexia, results from disrupted cellular proteostasis characterized by decreased protein synthesis, increased protein degradation, or some combination of both (Roy and Kumar, 2019). Testosterone and selective androgen receptor modulators (SARMS) have been suggested and studied as interventions against cancer induced wasting (Basaria et al., 2001; Bhasin et al., 2006; Narayanan et al., 2008; Li et al., 2021). Our group previously showed that the placebo treated group lost 3.3% lean body mass during the course of CAT while the testosterone treated group gained 3.2% (Wright et al., 2018). Testosterone treatment offered protection against the loss of lean mass involving a distinct set of genes compared to the CAT+P group. Cancer and associated therapies are associated with both skeletal muscle cachexia and concomitant cardiac atrophy and dysfunction (Kazemi-Bajestani et al., 2014; Murphy, 2016). Several proteomic shifts that occurred during CAT were alterations to pathways with known involvement in cardiomyopathy, and despite cardiac muscle being a distinct cell type from skeletal muscle, likely pointed towards similar myopathic processes in skeletal muscle of these patients (Rausch et al., 2021). Nevertheless, it should be noted that an additional ancillary analysis of this study demonstrated that testosterone treatment was associated with improvement in some measures of cardiac function including left ventricular ejection fraction (Scott et al., 2019).

The observed decrease in contractile proteins MYL3 and MYL1 in the CAT+P group are consistent with cachectic myopathy. However, the structural protein DES increased in CAT+P, which is counterintuitive to the catabolic phenotype of these patients. Expression of the intermediate filament DES has been shown to increase near carcinomatous glands and microvessels but it is difficult to explain the increase in expression within skeletal muscle observed here (Arentz et al., 2011; Mittal, 2020). It is also important to consider the potential contribution of standard of care (SOC) treatment on PRE to POST treatment changes. Muscle wasting can be synergistically driven by factors related to cancer-host interactions as well as toxicities contributed by CAT (Campelj et al., 2021). Chemotherapy can induce increased DES expression in rhabdomyosarcomas (Klunder et al., 2003) and it is expected that the SOC that was offered to the patients altered protein expression and post-translational modifications to skeletal muscle proteoforms over the course of treatment in this investigation.

The stretch-responsive ankyrin-repeat protein ANKRD2 (Kojic et al., 2011) increased along with lean body mass in response to CAT+T. ANKRD2 has been associated with increased hypertrophy (Kemp et al., 2000) which could be driven by a testosterone induced stimulation of the IGF-I signaling system (Urban et al., 1995). ANKRD2 is also a potent repressor of inflammatory responses through direct interaction with the NF-κB repressor subunit p50. ANKRD2 protein and phosphorylation levels are shown to modulate the balance between physiological and pathological inflammatory responses in muscle (Bean et al., 2014). The observation of testosterone induced upregulation of ANKRD2 also adds support to the anti-inflammatory properties of testosterone. There is evidence that the expression of the structural and mechanosensitive proteins DES and ANKRD1 (which is expressed in cardiac and smooth muscle, but not skeletal muscle) are inversely regulated in smooth muscle (Mohamed and Boriek, 2012). The mechanisms between DES and ANKRD2 expression in skeletal muscle are unclear but such an inverse relationship could further support the observed increases in DES in the placebo group vs. increased ANKRD2 in testosterone treated patients.

Protein functions can be mediated through reversible post-translational modifications such as phosphorylation or S-nitrosylation of specific protein sites. S-nitrosylation involves the post-translational modification of proteins through covalent attachment of a nitrogen monoxide group to the thiol side chain of a cysteine (S) amino acid within the peptide. Nitric oxide (NO) necessary for S-nitrosylation is produced by nitric oxide synthase (NOS) (Foster et al., 2009). S-nitrosylation plays an important regulatory role in human physiology and alterations have been implicated in pathologies including cardiovascular, pulmonary, musculoskeletal and neurological (dys)function, as well as in cancer. NO-mediated protein S-nitrosylation has been established as a major signaling mechanism for cancer initiation and development (Tan et al., 2019). NO appears to have varying effects in cancer either being inhibitory or stimulatory depending on the case (Xu et al., 2002; Hickok and Thomas, 2010). NOS activity has been detected in tumor cells, and is associated with tumor grade, proliferation rate, etc. High levels of NOS expression may be cytostatic or cytotoxic for tumor cells, while low levels of NOS activity can have the opposite effect, resulting in tumor growth. Among the three known types of NOS, inducible NOS (iNOS) activity is upregulated in response to inflammatory cytokines (Ramamoorthy et al., 2009) and has been demonstrated in skeletal muscle (Foster et al., 2009; Hall et al., 2011). It is expected that much of the S-nitrosylation observed in the present study is the result of iNOS due to the inflammatory response to cancer (Hall et al., 2011). These results also support an anti-inflammatory role of testosterone treatment as more than double the number of proteoform S-nitrosylation changes occurred in response to CAT+P over the course of the study compared to CAT+T. Increased nitrosative stress is thought to be advantageous for tumor biology and interventions that reduce protein S-nitrosylation are actively being investigated as cancer treatment strategies (Rizza and Filomeni, 2020). Testosterone has been shown to inhibit iNOS synthesis (Friedl et al., 2000; Chavoshan et al., 2012). Additionally, as smooth muscle contraction was identified as one of the possible pathways affected by testosterone treatment it is possible that changes in endothelial NOS (eNOS) activity contributed to group differences in the levels of protein S-nitrosylation given the inevitability of vascular tissue in skeletal muscle homogenate. In the present study, 1.56 times as many proteoforms increased in S-nitrosylation compared to decreasing in CAT+P (36/23) while 1.45 times as many proteoforms decreased in S-nitrosylation compared to increasing in CAT+T. (16/11). The precise mechanism through which testosterone treatment may be regulating S-nitrosylation events deserves further investigation and whether there is therapeutic benefit for testosterone mediated denitrosylation in cancer therapy remains to be determined.

### Limitations

The primary objective of this randomized phase II study was to investigate whether testosterone administered during standard of care chemotherapy and/or radiation could help patients with squamous cell carcinoma maintain body weight, muscle size, and muscle strength during treatment. The patients were not classified on a cachectic scale, as described by Fearon et al. (Fearon et al., 2011). The timeframe of patients presenting to the clinic (and our study) was often very short and did not allow for proper classification while under observation, or the quantity of losses were not well known (even by the patient) before seeking treatment. Also, some patients were advanced or recurrent patients and weight loss had already occurred at an earlier stage. The authors also acknowledge the limited sample size and heterogeneity of the study population, including the range of ages, sex, cancer types, and treatments. This ancillary study was not powered to assess the contributions of age on treatment efficacy, although this is an acknowledged factor worth investigating (Horstman et al., 2015). As this was an intent-to-treat study within a population of squamous cell carcinoma patients, individual variations in treatments or daily activities of living were expected to occur. This included treatment interruptions and blood transfusions as directed by oncologists. Other factors besides CAT such as inactivity and poor nutrition could affect changes in protein expression during the observed treatment period. As previously reported, both placebo and testosterone treated patients remained consistently sedentary throughout treatment (vigorous activity below 0.5%) and were in general poor health and nutritional status (Wright et al., 2018). These confounding factors were similarly variable between treatment groups and were considered underlying conditions to CAT in context of the finding reported here.

As the analyses were targeted to skeletal muscle tissue, the authors decided against using murine specific background databases such as the murine based SysMyo Muscle Gene Set available in Enrichr or the Mus Musculus Gene Set available through GO Consortium that were muscle specific. We acknowledge that a consequence of comparing a human skeletal muscle proteome against more comprehensive databases such as KEGG and GO may be a biological bias, resulting in a ‘skeletal muscle signatures’ in the results (Timmons et al., 2015). However, the objective of this report was to compare the proteome in response to treatment without limiting the findings towards only previously identified skeletal muscle pathways. Despite the introduction of these muscle signatures, the results point towards differences in the signatures between treatment groups.

## Conclusion

Seven weeks of CAT was associated with changes in the skeletal muscle proteome of patients with cervical or head and neck cancer. Proteostasis was disrupted in patients receiving both adjunct testosterone and placebo, collectively consistent with cachectic myopathy, including loss of structural integrity, decreased contractile function, and disrupted metabolism. The two treatment groups differed in specific proteomic alterations to abundance, phosphorylation, and S-nitrosylation resulting in differences in altered pathways. In conjunction with prior results showing that adjunct testosterone treatment preserved skeletal muscle mass of patients, the differential proteomic changes in these two groups suggests that testosterone at least partially directed the functional pathways towards muscle tissue development and minimized the negative structural and contractile impact of cancer and associated therapies. The shotgun proteomic analysis approach revealed how CAT with or without testosterone treatment differentially affected protein expression and post-translational modification within skeletal muscle of these cancer patients. Targeted studies are necessary to determine the specific interactions and mechanisms that contribute to alterations in the proteome and how they lead to muscle wasting and other phenotypic changes associated with cancer cachexia.

## Data Availability

The datasets presented in this study can be found in online repositories. The names of the repository/repositories and accession number(s) can be found in the article/Supplementary Material.

## Supplementary Material

Supplementary Figure S1 GO Biological Prosesses. Proteins found to be altered in either abundance, phosphorylation, or S-nitrosylation were combined to identify affected gene sets using the 2021 GO Biological Process database.

Supplementary Figure S2 KEGG Pathways. Proteins found to be altered in either abundance, phosphorylation, or S-nitrosylation were combined to identify affected pathways using the 2021 KEGG pathway database.

Supplementary Table S1 Protein abundances (A) CAT+P (B) CAT+T.

Supplementary Table S2 Protein Phosphorylation (A) CAT+P (B) CAT+T.

Supplementary Table S3 Protein S-Nitrosylation (A) CAT+P (B) CAT+T.
