# Supplementary figures and images for: Cancer and Associated Therapies Impact the Skeletal Muscle Proteome
Source: Front Physiol. 2022 May 27;13:879263. doi: 10.3389/fphys.2022.879263 (PMC9184684; doi:10.3389/fphys.2022.879263)

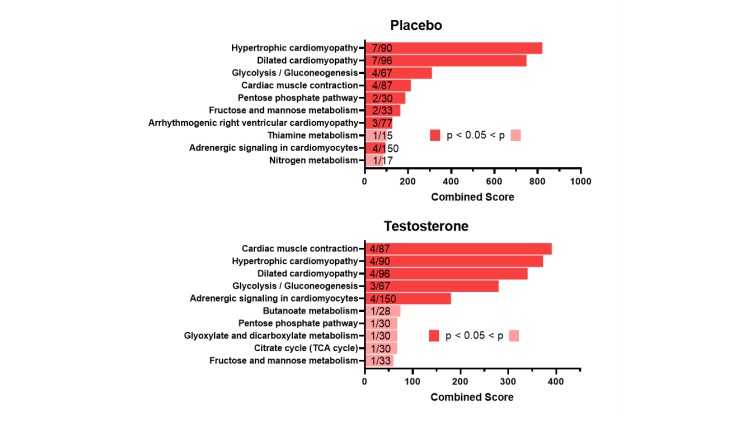

Supplement: Supplementary file 1 [file Image2.jpg]

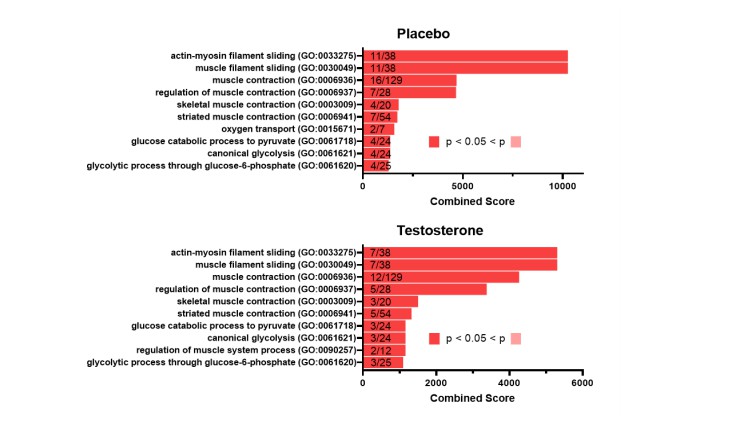

Supplement: Supplementary file 5 [file Image1.jpg]
