# Supplementary material for: Cancer and Associated Therapies Impact the Skeletal Muscle Proteome
Source: Front Physiol. 2022 May 27;13:879263. doi: 10.3389/fphys.2022.879263 (PMC9184684; doi:10.3389/fphys.2022.879263)
Supplement: Supplementary file 2 [file Table1.docx]

S1A. Protein Abundances - CAT+P
Based on pre to post changes of the following proteoforms: ANXA2, DES, IGKC, MYL1, MYL3, S100A6, VCL.
KEGG 2021 Human
Index	Name	Genes	Overlap	P-value	Adjusted p-value	Odds Ratio	Combined score
1	Hypertrophic cardiomyopathy	DES;MYL3	2/90	0.0004144	0.003300	90.48	704.70
2	Dilated cardiomyopathy	DES;MYL3	2/96	0.0004714	0.003300	84.68	648.61
3	Adherens junction	VCL	1/71	0.02459	0.06865	47.44	175.77
4	Bacterial invasion of epithelial cells	VCL	1/77	0.02664	0.06865	43.68	158.34
5	Arrhythmogenic right ventricular cardiomyopathy	DES	1/77	0.02664	0.06865	43.68	158.34
6	Cardiac muscle contraction	MYL3	1/87	0.03006	0.06865	38.58	135.20
7	Amoebiasis	VCL	1/102	0.03516	0.06865	32.83	109.89
8	Leukocyte transendothelial migration	VCL	1/114	0.03923	0.06865	29.32	94.95
9	Apelin signaling pathway	MYL3	1/137	0.04698	0.07188	24.33	74.41
10	Adrenergic signaling in cardiomyocytes	MYL3	1/150	0.05134	0.07188	22.20	65.91

GO Biological Process 2021
Index	Name	Genes	Overlap	P-value	Adjusted p-value	Odds Ratio	Combined score
1	actin-myosin filament sliding (GO:0033275)	DES;MYL1;MYL3;VCL	4/129	2.203e-7	0.000006021	427.67	6555.48
2	muscle filament sliding (GO:0030049)	DES;MYL1;MYL3	3/38	2.203e-7	0.000006021	427.67	6555.48
3	positive regulation of low-density lipoprotein receptor activity (GO:1905599)	DES;MYL1;MYL3	3/38	0.001749	0.01791	832.88	5287.72
4	regulation of low-density lipoprotein particle receptor catabolic process (GO:0032803)	ANXA2	01/05	0.001749	0.01791	832.88	5287.72
5	positive regulation of lipoprotein particle clearance (GO:0010986)	ANXA2	01/05	0.002098	0.01791	666.27	4108.59
6	positive regulation of receptor binding (GO:1900122)	ANXA2	01/06	0.002098	0.01791	666.27	4108.59
7	membrane raft assembly (GO:0001765)	ANXA2	01/06	0.002098	0.01791	666.27	4108.59
8	muscle contraction (GO:0006936)	ANXA2	01/06	5.695e-8	0.000004670	211.93	3535.16
9	positive regulation of vesicle fusion (GO:0031340)	ANXA2	01/07	0.002448	0.01791	555.19	3338.15
10	bone cell development (GO:0098751)	ANXA2	01/09	0.003146	0.01791	416.35	2398.85


Table S1B. Protein Abundances - CAT+T
Based on pre to post changes of  the following proteoforms: ACO2, ACTG2, ANKRD2, BCORP1, BEST3, MIB2, MYH7. 
KEGG 2021 Human
Index	Name	Genes	Overlap	P-value	Adjusted p-value	Odds Ratio	Combined score
1	Glyoxylate and dicarboxylate metabolism	ACO2	01/30	0.01045	0.05750	114.74	523.28
2	Citrate cycle (TCA cycle)	ACO2	01/30	0.01045	0.05750	114.74	523.28
3	Viral myocarditis	MYH7	1/60	0.02081	0.06073	56.31	218.04
4	Cardiac muscle contraction	MYH7	1/87	0.03006	0.06073	38.58	135.20
5	Hypertrophic cardiomyopathy	MYH7	1/90	0.03108	0.06073	37.27	129.38
6	Dilated cardiomyopathy	MYH7	1/96	0.03312	0.06073	34.91	118.95
7	Thyroid hormone signaling pathway	MYH7	1/121	0.04159	0.06272	27.60	87.77
8	Vascular smooth muscle contraction	ACTG2	1/133	0.04564	0.06272	25.08	77.41
9	Adrenergic signaling in cardiomyocytes	MYH7	1/150	0.05134	0.06272	22.20	65.91
10	cGMP-PKG signaling pathway	MYH7	1/167	0.05701	0.06272	19.91	57.02

GO Biological Process 2021
Index	Name	Genes	Overlap	P-value	Adjusted p-value	Odds Ratio	Combined score
1	regulation of transcription from RNA polymerase II promoter in response to oxidative stress (GO:0043619)	ANKRD2	01/08	0.002797	0.03088	475.86	2797.66
2	adult heart development (GO:0007512)	MYH7	01/09	0.003146	0.03088	416.35	2398.85
3	tricarboxylic acid metabolic process (GO:0072350)	ACO2	01/12	0.004193	0.03088	302.76	1657.40
4	negative regulation of myoblast differentiation (GO:0045662)	ANKRD2	01/13	0.004542	0.03088	277.51	1497.03
5	muscle contraction (GO:0006936)	ANKRD2;ACTG2;MYH7	3/129	0.000009003	0.0003061	118.26	1373.89
6	skeletal muscle contraction (GO:0003009)	MYH7	01/20	0.006980	0.03459	175.21	869.87
7	ventricular cardiac muscle tissue development (GO:0003229)	MYH7	01/25	0.008718	0.03459	138.67	657.63
8	cardiac muscle tissue morphogenesis (GO:0055008)	MYH7	1/32	0.01115	0.03459	107.32	482.58
9	regulation of myoblast differentiation (GO:0045661)	ANKRD2	1/34	0.01184	0.03459	100.81	447.20
10	ventricular cardiac muscle tissue morphogenesis (GO:0055010)	MYH7	1/34	0.01184	0.03459	100.81	447.20
