# Supplementary material for: Cancer and Associated Therapies Impact the Skeletal Muscle Proteome
Source: Front Physiol. 2022 May 27;13:879263. doi: 10.3389/fphys.2022.879263 (PMC9184684; doi:10.3389/fphys.2022.879263)
Supplement: Supplementary file 3 [file Table2.docx]

Table S2A. Protein Phosphorylation - CAT+P 
Based on pre to post changes of the following proteoforms: ACSM2B, ACTA1, ACTG2, CKM, MB, YWHAG. 
KEGG 2021 Human
Index	Name	Genes	Overlap	P-value	Adjusted p-value	Odds Ratio	Combined score
1	Butanoate metabolism	ACSM2B	01/28	0.008372	0.06161	147.90	707.41
2	Arginine and proline metabolism	CKM	1/50	0.01491	0.06161	81.41	342.39
3	Cell cycle	YWHAG	1/124	0.03663	0.06161	32.31	106.85
4	Oocyte meiosis	YWHAG	1/129	0.03809	0.06161	31.04	101.44
5	Vascular smooth muscle contraction	ACTG2	1/133	0.03925	0.06161	30.09	97.44
6	Hepatitis C	YWHAG	1/157	0.04619	0.06161	25.43	78.21
7	Hippo signaling pathway	YWHAG	1/163	0.04792	0.06161	24.48	74.39
8	Viral carcinogenesis	YWHAG	1/203	0.05938	0.06681	19.60	55.33
9	PI3K-Akt signaling pathway	YWHAG	1/354	0.1016	0.1016	11.13	25.44

GO Biological Process 2021
Index	Name	Genes	Overlap	P-value	Adjusted p-value	Odds Ratio	Combined score
1	skeletal muscle thin filament assembly (GO:0030240)	ACTA1	01/06	0.001799	0.02112	799.56	5053.71
2	oxygen transport (GO:0015671)	MB	01/07	0.002098	0.02112	666.27	4108.59
3	creatine metabolic process (GO:0006600)	CKM	01/07	0.002098	0.02112	666.27	4108.59
4	skeletal myofibril assembly (GO:0014866)	ACTA1	01/08	0.002398	0.02112	571.06	3445.29
5	skeletal muscle fiber development (GO:0048741)	ACTA1	01/09	0.002697	0.02112	499.65	2955.69
6	myotube cell development (GO:0014904)	ACTA1	01/11	0.003296	0.02112	399.68	2284.21
7	gas transport (GO:0015669)	MB	01/12	0.003595	0.02112	363.33	2044.88
8	regulation of protein insertion into mitochondrial membrane involved in apoptotic signaling pathway (GO:1900739)	YWHAG	01/26	0.007776	0.02667	159.75	775.88
9	positive regulation of protein insertion into mitochondrial membrane involved in apoptotic signaling pathway (GO:1900740)	YWHAG	01/26	0.007776	0.02667	159.75	775.88
10	purine nucleoside bisphosphate metabolic process (GO:0034032)	ACSM2B	01/31	0.009265	0.02667	133.09	623.08


Table S2B. Protein Phosphorylation - CAT+T
Based on pre to post changes of the following proteoforms: ACTG2, ANKRD2, ARHGEF25, BCORP1, MACF1, MYL1. 
KEGG 2021 Human
Index	Name	Genes	Overlap	P-value	Adjusted p-value	Odds Ratio	Combined score
1	Vascular smooth muscle contraction	ACTG2	1/133	0.03925	0.03925	30.09	97.44


GO Biological Process 2021
Index	Name	Genes	Overlap	P-value	Adjusted p-value	Odds Ratio	Combined score
1	regulation of transcription from RNA polymerase II promoter in response to oxidative stress (GO:0043619)	ANKRD2	01/08	0.002398	0.03026	571.06	3445.29
2	muscle contraction (GO:0006936)	MYL1;ANKRD2;ACTG2	3/129	0.000005169	0.0002068	157.68	1919.44
3	negative regulation of myoblast differentiation (GO:0045662)	ANKRD2	01/13	0.003894	0.03026	333.03	1847.77
4	regulation of cell projection organization (GO:0031344)	MACF1	01/14	0.004193	0.03026	307.40	1682.80
5	regulation of neuron projection arborization (GO:0150011)	MACF1	01/15	0.004492	0.03026	285.43	1542.87
6	regulation of cell-substrate junction assembly (GO:0090109)	MACF1	01/21	0.006284	0.03026	199.74	1012.63
7	positive regulation of axon extension (GO:0045773)	MACF1	01/22	0.006583	0.03026	190.22	955.53
8	Golgi to plasma membrane protein transport (GO:0043001)	MACF1	01/30	0.008967	0.03026	137.69	649.09
9	regulation of cell morphogenesis (GO:0022604)	MACF1	1/32	0.009563	0.03026	128.79	598.87
10	regulation of myoblast differentiation (GO:0045661)	ANKRD2	1/34	0.01016	0.03026	120.98	555.22


an
Index	Name	Genes	Overlap	P-value	Adjusted p-value	Odds Ratio	Combined score
1	Hypertrophic cardiomyopathy	MYL2;TPM1;EMD;ACTG1;MYH7	5/90	0.000001088	0.00004722	32.47	445.92
2	Dilated cardiomyopathy	MYL2;TPM1;EMD;ACTG1;MYH7	5/96	0.000001499	0.00004722	30.32	406.66
3	Glycolysis / Gluconeogenesis	PKM;ALDOC;ENO1;ALDOA	4/67	0.00001061	0.0002228	34.14	391.05
4	Pentose phosphate pathway	ALDOC;ALDOA	2/30	0.001720	0.01806	36.50	232.36
5	Fructose and mannose metabolism	ALDOC;ALDOA	2/33	0.002079	0.01872	32.97	203.59
6	Cardiac muscle contraction	MYL2;TPM1;MYH7	3/87	0.0007521	0.01184	18.68	134.36
7	Thiamine metabolism	AK1	01/15	0.03032	0.1274	35.62	124.51
8	Nitrogen metabolism	CA3	01/17	0.03430	0.1350	31.16	105.10
9	HIF-1 signaling pathway	ALDOC;ENO1;ALDOA	3/109	0.001444	0.01806	14.79	96.71
10	Viral myocarditis	ACTG1;MYH7	2/60	0.006732	0.04712	17.60	88.00

 GO Biological Process 2021
Index	Name	Genes	Overlap	P-value	Adjusted p-value	Odds Ratio	Combined score
1	actin-myosin filament sliding (GO:0033275)	ACTA1;TNNT1;MYL2;TPM1;TNNT3;TNNI1;MYH4;MYH7	8/38	7.048e-15	8.505e-13	161.04	5247.72
2	muscle filament sliding (GO:0030049)	ACTA1;TNNT1;MYL2;TPM1;TNNT3;TNNI1;MYH4;MYH7	8/38	7.048e-15	8.505e-13	161.04	5247.72
3	regulation of muscle contraction (GO:0006937)	TNNT1;MYL2;TPM1;TNNT3;TNNI1;ENO1	06/28	1.845e-11	1.669e-9	155.35	3839.74
4	muscle contraction (GO:0006936)	ACTA1;TNNT1;MYL2;TPM1;TNNT3;TNNI1;ANKRD2;ALDOA;MYH4;EMD;ACTG2;MYH7	12/129	2.070e-17	7.495e-15	70.18	2695.86
5	skeletal muscle contraction (GO:0003009)	TNNT1;TNNT3;TNNI1;MYH7	04/20	7.189e-8	0.000003718	134.75	2216.39
6	oxygen transport (GO:0015671)	MB;HBB	02/07	0.00008554	0.001346	204.66	1916.91
7	glucose catabolic process to pyruvate (GO:0061718)	PKM;ALDOC;ENO1;ALDOA	04/24	1.567e-7	0.000006304	107.78	1688.75
8	canonical glycolysis (GO:0061621)	PKM;ALDOC;ENO1;ALDOA	04/24	1.567e-7	0.000006304	107.78	1688.75
9	glycolytic process through glucose-6-phosphate (GO:0061620)	PKM;ALDOC;ENO1;ALDOA	04/25	1.863e-7	0.000006744	102.64	1590.51
10	fructose metabolic process (GO:0006000)	ALDOC;ALDOA	02/08	0.0001139	0.001586	170.54	1548.51
