# Supplementary material for: Cancer and Associated Therapies Impact the Skeletal Muscle Proteome
Source: Front Physiol. 2022 May 27;13:879263. doi: 10.3389/fphys.2022.879263 (PMC9184684; doi:10.3389/fphys.2022.879263)
Supplement: Supplementary file 4 [file Table3.docx]

Table S3A. Protein S-Nitrosylation - CAT+P
Based on pre to post changes of the following proteoforms: ACO2, ACSM2B, ACTA1, ACTG1, ACTG2, AK1, ALDOA, ALDOC, ANKRD2, ANXA2, ANXA5, BCORP1, BEST3, C12orf43, CA3, CCNL2, CKM, DCAF11, EEF1A2, EMD, ENO1, GPATCH8, HBB, HBD, KRT10, LDB3, MACF1, MB, MYH4, MYH7, MYL2, MYOZ1, NEFL, NR1H2, PKM,  SKA2, SLC25A47, TNNI1, TNNT1, TNNT3, TPM1. 
KEGG 2021 Human
Index	Name	Genes	Overlap	P-value	Adjusted p-value	Odds Ratio	Combined score
1	Hypertrophic cardiomyopathy	MYL2;TPM1;EMD;ACTG1;MYH7	5/90	0.000001088	0.00004722	32.47	445.92
2	Dilated cardiomyopathy	MYL2;TPM1;EMD;ACTG1;MYH7	5/96	0.000001499	0.00004722	30.32	406.66
3	Glycolysis / Gluconeogenesis	PKM;ALDOC;ENO1;ALDOA	4/67	0.00001061	0.0002228	34.14	391.05
4	Pentose phosphate pathway	ALDOC;ALDOA	2/30	0.001720	0.01806	36.50	232.36
5	Fructose and mannose metabolism	ALDOC;ALDOA	2/33	0.002079	0.01872	32.97	203.59
6	Cardiac muscle contraction	MYL2;TPM1;MYH7	3/87	0.0007521	0.01184	18.68	134.36
7	Thiamine metabolism	AK1	01/15	0.03032	0.1274	35.62	124.51
8	Nitrogen metabolism	CA3	01/17	0.03430	0.1350	31.16	105.10
9	HIF-1 signaling pathway	ALDOC;ENO1;ALDOA	3/109	0.001444	0.01806	14.79	96.71
10	Viral myocarditis	ACTG1;MYH7	2/60	0.006732	0.04712	17.60	88.00

 GO Biological Process 2021
Index	Name	Genes	Overlap	P-value	Adjusted p-value	Odds Ratio	Combined score
1	actin-myosin filament sliding (GO:0033275)	ACTA1;TNNT1;MYL2;TPM1;TNNT3;TNNI1;MYH4;MYH7	8/38	7.048e-15	8.505e-13	161.04	5247.72
2	muscle filament sliding (GO:0030049)	ACTA1;TNNT1;MYL2;TPM1;TNNT3;TNNI1;MYH4;MYH7	8/38	7.048e-15	8.505e-13	161.04	5247.72
3	regulation of muscle contraction (GO:0006937)	TNNT1;MYL2;TPM1;TNNT3;TNNI1;ENO1	06/28	1.845e-11	1.669e-9	155.35	3839.74
4	muscle contraction (GO:0006936)	ACTA1;TNNT1;MYL2;TPM1;TNNT3;TNNI1;ANKRD2;ALDOA;MYH4;EMD;ACTG2;MYH7	12/129	2.070e-17	7.495e-15	70.18	2695.86
5	skeletal muscle contraction (GO:0003009)	TNNT1;TNNT3;TNNI1;MYH7	04/20	7.189e-8	0.000003718	134.75	2216.39
6	oxygen transport (GO:0015671)	MB;HBB	02/07	0.00008554	0.001346	204.66	1916.91
7	glucose catabolic process to pyruvate (GO:0061718)	PKM;ALDOC;ENO1;ALDOA	04/24	1.567e-7	0.000006304	107.78	1688.75
8	canonical glycolysis (GO:0061621)	PKM;ALDOC;ENO1;ALDOA	04/24	1.567e-7	0.000006304	107.78	1688.75
9	glycolytic process through glucose-6-phosphate (GO:0061620)	PKM;ALDOC;ENO1;ALDOA	04/25	1.863e-7	0.000006744	102.64	1590.51
10	fructose metabolic process (GO:0006000)	ALDOC;ALDOA	02/08	0.0001139	0.001586	170.54	1548.51


Table S3B. Protein S-Nitrosylation - CAT+T
Based on pre to post changes of the following proteoforms: ACSM2B, ACTG2, ALDOA, ANKRD2, C12orf43, ENO1, HBA2, KRT1, KRT10, LDB3, MYL2, MYLPF, NEFL, NR1H2, MYLPF, PKM, PPIAL4D, PYGM, SKA2, TNNC2, TNNT1, TPM1, TPM2, TRIM72. 
KEGG 2021 Human
Index	Name	Genes	Overlap	P-value	Adjusted p-value	Odds Ratio	Combined score
1	Glycolysis / Gluconeogenesis	PKM;ENO1;ALDOA	3/67	0.00006915	0.001865	44.45	425.76
2	Cardiac muscle contraction	TPM2;MYL2;TPM1	3/87	0.0001506	0.001865	33.83	297.73
3	Hypertrophic cardiomyopathy	TPM2;MYL2;TPM1	3/90	0.0001665	0.001865	32.66	284.14
4	Dilated cardiomyopathy	TPM2;MYL2;TPM1	3/96	0.0002016	0.001865	30.54	259.89
5	Adrenergic signaling in cardiomyocytes	TPM2;MYL2;TPM1	3/150	0.0007455	0.005517	19.27	138.77
6	Butanoate metabolism	ACSM2B	01/28	0.03308	0.08780	32.12	109.50
7	Pentose phosphate pathway	ALDOA	01/30	0.03541	0.08780	29.91	99.91
8	Fructose and mannose metabolism	ALDOA	1/33	0.03888	0.08940	27.10	87.99
9	Glucagon signaling pathway	PKM;PYGM	2/107	0.007248	0.03367	17.20	84.77
10	Insulin resistance	NR1H2;PYGM	2/108	0.007379	0.03367	17.04	83.66


GO Biological Process 2021
Index	Name	Genes	Overlap	P-value	Adjusted p-value	Odds Ratio	Combined score
1	regulation of muscle contraction (GO:0006937)	TNNT1;MYL2;TNNC2;TPM1;ENO1	05/28	1.539e-10	1.762e-8	228.30	5158.33
2	muscle contraction (GO:0006936)	MYLPF;TRIM72;TNNT1;TPM2;MYL2;TNNC2;TPM1;ANKRD2;ALDOA;ACTG2	10/129	1.587e-16	3.633e-14	119.19	4336.09
3	actin-myosin filament sliding (GO:0033275)	TNNT1;TPM2;MYL2;TNNC2;TPM1	5/38	7.796e-10	4.463e-8	159.04	3335.32
4	muscle filament sliding (GO:0030049)	TNNT1;TPM2;MYL2;TNNC2;TPM1	5/38	7.796e-10	4.463e-8	159.04	3335.32
5	glucose catabolic process to pyruvate (GO:0061718)	PKM;ENO1;ALDOA	03/24	0.000003022	0.00009823	135.75	1725.29
6	canonical glycolysis (GO:0061621)	PKM;ENO1;ALDOA	03/24	0.000003022	0.00009823	135.75	1725.29
7	regulation of muscle system process (GO:0090257)	TNNC2;TPM1	02/12	0.00009042	0.001479	181.51	1690.05
8	glycolytic process through glucose-6-phosphate (GO:0061620)	PKM;ENO1;ALDOA	03/25	0.000003432	0.00009823	129.57	1630.33
9	protein heterotetramerization (GO:0051290)	KRT1;KRT10	02/13	0.0001068	0.001630	165.00	1508.89
10	glycolytic process (GO:0006096)	PKM;ENO1;ALDOA	03/29	0.000005435	0.0001383	109.62	1328.83
